# Selenium Supplementation Affects Physiological and Biochemical Processes to Improve Fodder Yield and Quality of Maize (*Zea mays* L.) under Water Deficit Conditions

**DOI:** 10.3389/fpls.2016.01438

**Published:** 2016-09-27

**Authors:** Fahim Nawaz, Muhammad Naeem, Muhammad Y. Ashraf, Muhammad N. Tahir, Bilal Zulfiqar, Muhammad Salahuddin, Rana N. Shabbir, Muhammad Aslam

**Affiliations:** ^1^Department of Agronomy, Muhammad Nawaz Sharif University of AgricultureMultan, Pakistan; ^2^Department of Agronomy, UCA and ES, The Islamia University of BahawalpurBahawalpur, Pakistan; ^3^University College of Veterinary and Animal Sciences, The Islamia University of BahawalpurBahawalpur, Pakistan; ^4^Nuclear Institute for Agriculture and BiologyFaisalabad, Pakistan; ^5^Department of Agronomy, Agriculture College, Bahauddin Zakariya UniversityMultan, Pakistan

**Keywords:** Se, drought stress, photosynthetic pigments, antioxidant enzymes, fodder quality, maize

## Abstract

Climate change is one of the most complex challenges that pose serious threats to livelihoods of poor people who rely heavily on agriculture and livestock particularly in climate-sensitive developing countries of the world. The negative effects of water scarcity, due to climate change, are not limited to productivity food crops but have far-reaching consequences on livestock feed production systems. Selenium (Se) is considered essential for animal health and has also been reported to counteract various abiotic stresses in plants, however, understanding of Se regulated mechanisms for improving nutritional status of fodder crops remains elusive. We report the effects of exogenous selenium supply on physiological and biochemical processes that may influence green fodder yield and quality of maize (*Zea mays* L.) under drought stress conditions. The plants were grown in lysimeter tanks under natural conditions and were subjected to normal (100% field capacity) and water stress (60% field capacity) conditions. Foliar spray of Se was carried out before the start of tasseling stage (65 days after sowing) and was repeated after 1 week, whereas, water spray was used as a control. Drought stress markedly reduced the water status, pigments and green fodder yield and resulted in low forage quality in water stressed maize plants. Nevertheless, exogenous Se application at 40 mg L^-1^ resulted in less negative leaf water potential (41%) and enhanced relative water contents (30%), total chlorophyll (53%), carotenoid contents (60%), accumulation of total free amino acids (40%) and activities of superoxide dismutase (53%), catalase (30%), peroxidase (27%), and ascorbate peroxidase (27%) with respect to control under water deficit conditions. Consequently, Se regulated processes improved fodder yield (15%) and increased crude protein (47%), fiber (10%), nitrogen free extract (10%) and Se content (36%) but did not affect crude ash content in water stressed maize plants. We propose that Se foliar spray (40 mg L^-1^) is a handy, feasible and cost-effective approach to improve maize fodder yield and quality in arid and semi-arid regions of the world facing acute shortage of water.

## Introduction

Livestock is a major livelihood for poor people in developing countries fulfilling their social, economic and risk management functions. Climate change has increased the vulnerability of livestock systems, which may trigger a loss of livelihoods of poor communities in arid and semi-arid regions of the world. Increase in temperatures and changes in rainfall patterns would heighten the risks of existing vector-borne diseases accompanied by the emergence and spread of new diseases. Non-availability of water would severely hamper the livestock feed production systems in nomadic and desert regions of the world where people are solely dependent on livestock sector for their survival.

Drought stress negatively influences the nutritive value of forages by affecting sugar, crude fiber, protein, total ash contents and nitrogen free extracts (Bibi et al., 2012; Küchenmeister et al., 2013). Identification of an effective stress ameliorant for improving yield and quality of fodder crops under water deficit conditions is essential in today’s changing climates. Selenium (Se) is not considered essential for plant growth but recent reports indicate its beneficial role in plants exposed to various abiotic stresses such as salinity (Abul-Soud and Abd-Elrahman, 2016), high temperature (Balal et al., 2016), chilling injury (Hawrylak-Nowak et al., 2010; Akladious, 2012), metals accumulation (Li et al., 2016), UV-B induced oxidative damage (Yao et al., 2011) and drought stress (Nawaz et al., 2015a,b). Se mediated enhancement in plant resistance/tolerance to environmental stresses is attributed to its positive role in several physiological and biochemical mechanisms such as maintenance of water status (Djanaguiraman et al., 2005), enhancement in pigments (Malik et al., 2012), regulation of photosynthetic machinery (Balal et al., 2016), accumulation of osmoprotectants (Akbulut and Çakir, 2010) and activation of antioxidant enzymes (Hasanuzzaman et al., 2012; Ahmad et al., 2016). Moreover, it has also been reported to improve yield of food crops like wheat (Broadley et al., 2010; Nawaz et al., 2015b), barley (Ducsay et al., 2009), rice (Wang et al., 2013), and maize (Chilimba et al., 2012). However, the understanding of physiological and biochemical mechanisms that underlie the positive effects of Se in improving yield and quality of fodders under water deficit conditions remains elusive.

Se is an integral constituent of the glutathione peroxidase family (GSH-Px) and is considered essential for humans (Leccia et al., 1993) and animals (Hefnawy and Tórtora-Pérez, 2010) due to its antioxidant, anticancer, and antivirus properties. It stimulates growth and enhances resistance to diseases in animals, being involved in the production of antibodies and in the killing of microorganisms engulfed by macrophages (Khanal and Knight, 2010). Se deficiency in livestock is associated with reduced growth, appetite, muscular activity and reproductive fertility (Fordyce, 2013), whereas excessive Se intake (>1–5 mg Se kg^-1^ dry matter) may also result in hair loss, hoof deformation, lack of vitality and respiratory failure (Dhillon and Dhillon, 2003; White, 2016). Se intake by animals is directly related to the amount of Se taken up by the plants (Fordyce, 2005), which is linked to Se phytoavailability in the soil (White, 2016). Biofortication of fodder crops with Se is essential for the development of balanced mineral ration for livestock because many pastures are naturally low in Se levels (Žáková, 2014) particularly during spring season hence Se-fortified commercial feed additives are used to maintain the physiological Se levels in blood (Drahoňovský et al., 2016) for the prevention of Se deficiency related diseases like white muscle and reproductive infertility in cattle and sheep.

The information concerning the effects of exogenous Se supply on Se accumulation and nutritive value of forages under drought stress is limited. Here, we hypothesize that Se foliar spray influences physiological and biochemical processes to improve fodder yield and quality of maize under water deficit conditions.

## Materials and Methods

### Crop Husbandry and Experimental Layout

The experiments were carried out in 1 m deep lysimeter tanks (3 m length × 3 m dia) separated by a buffer zone of 15 cm thick-cemented wall on each side to prevent seepage losses. The lysimeters were fitted with a manually operated moveable, light transmissive rain-out shelter. Precision leveling was done before sowing to ensure even distribution of water. The soil samples arbitrarily collected from three different sites of each tank were used to determine the physiochemical characteristics of the soil according to the method of Jackson (1962) with results as follows: soil texture = sandy loam; pH = 7.89; saturation percentage = 32.11%; electrical conductivity = 0.97 dS m^-1^; soil organic matter = 0.87%; available phosphorous (P) = 10.39 mg kg^-1^; potassium (K) = 68 mg kg^-1^ and nitrogen (N) = 347 mg kg^-1^. Total Se content in the soil was determined following the procedure described by Ramos et al. (2010) and was found to be 0.074 mg kg^-1^. Plant nutrient requirements of N, P, and K were met by the application of recommended doses of urea (250 kg ha^-1^), diammonium phosphate (140 kg ha^-1^) and sulfate of potash (150 kg ha^-1^). All P, K, and 1/8th N was mixed the surface layer (0–15 cm) before sowing, whereas 1/5th N was applied at V2 and the remaining N was broadcasted in two equal splits at V12 and V14 growth stages of maize.

The seeds of indigenous maize cultivar (*Zea mays* L. cv. Pak-Afghoi) were obtained from Regional Agricultural Research Institute (RARI), Bahawalpur and were disinfected with recommended doses of Topsin-M 70 WP (2–2.5 g kg^-1^ seed) and Imidacloprid (4 g kg^-1^ seed) prior to sowing. The seeds were hand drilled in rows of 0.80 m length keeping P × P and R × R distance of 0.20 and 0.70 m, respectively when soil was at field capacity (FC) condition. The weeds were uprooted manually whenever found necessary. The experiment was laid out in a 2 × 2 factorial scheme and experimental setup consisted of four treatments: normal irrigations with water spray (N), Se foliar spray under normal conditions (Se), drought stress with water spray (D) and Se foliar under drought stress conditions (D+Se) with three repeats. The plants were harvested 1 week after the second foliar treatment and the youngest, fully expanded fresh leaves were collected before harvesting for the determination of physiological and biochemical attributes in maize.

### Drought Stress and Se Treatments

Drought stress treatments, i.e., normal water supply (100% FC) and water stress (60% FC) were allocated separately to each tank in the lysimeter. The normal plants received approximately 790 mm tap water (an irrigation of 75–80 mm per week), whereas water stressed plants received approximately 500 mm irrigation water (about 48–50 mm irrigation applied per week) till harvesting. A water meter fixed at the water supply equipment was used to estimate the quantity of applied irrigations.

Se foliar treatment of 40 mg Se L^-1^ was developed using sodium selenate (Na_2_SeO_4_; MW = 188.95; purity ≥ 98.0%; Sigma-Aldrich, St. Louis, MO, USA) and was later verified analytically using atomic absorption spectrometry technique (Krishnaiah et al., 2003). Se foliar spray, containing 0.1% Tween-20, was carried out before the onset of tasseling stage (65 days after sowing) and was repeated after 1 week. Water spray, containing the same amount of Tween-20 (0.1%), was used as a control. The spraying was performed with a compression layer of 1 L capacity and was carried out early in the morning (between 06:00 and 08:00 a.m.). The plants were harvested at late vegetative stage (V16-17) to determine green fodder yield (only first cutting yield was taken).

### Measurement of Leaf Water Status

The fully expanded youngest, fresh leaf collected for each treatment was used for the estimation of leaf water potential (ψ_w_) using “Scholander” type pressure chamber Model 1000 (PMS, Oregon-USA). The same leaf was weighed immediately afterward to record fresh weight (FW) and then dipped in distilled water for 24 h at 4°C. The leaves were then taken out, wiped with a tissue paper and the turgid weight (TW) was recorded. For dry weight (DW) determination, the samples were kept in an oven at 65°C for 72 h. Leaf relative water contents (RWCs) were calculated according to the following formula reported by Mayak et al. (2004).

$$\begin{array}{c} \mathrm{RWC}\;=\;[(FW-DW)/(TW-DW)]\;\times \;100. \end{array}$$

The excised leaf water loss (ELWL) was also estimated on youngest leaves, which were excised and weighed immediately to record FW. The leaves were then incubated for 6 h at 28°C and 50% relative humidity to record incubation weight (IW) and later put in an oven at 65°C for 72 h to estimate DW. The following formula proposed by Clarke and McCaig (1982) was used to calculate ELWL.

$$\begin{array}{c} \mathrm{ELWL}\;=\;(FW-IW)/(FW-DW)\;\times \;100. \end{array}$$

For the determination of excised leaf water retention (ELWR), the youngest leaves collected for each treatment were weighed to record FW, kept at room temperature (25°C) for 6 h and reweighed (WL). ELWR was calculated using the following formula suggested by Lonbani and Arzani (2011).

$$\begin{array}{c} \mathrm{ELWR}\;=\;(FW-WL)/FW\;\times \;100. \end{array}$$

### Estimation of Pigments

Fresh leaf material (1.0 g) collected for each treatment was chopped into 0.5 cm segments and later extracted in 10 mL acetone (80%) at 4°C over-night for the estimation of chlorophyll (Chl) and carotenoid (Car) contents according to the methods of Arnon (1949) and Davies (1976). Following formulae were used to calculate Chl_a_, Chl_b_, total Chl and CAR contents after measuring the absorbance of supernatant on a spectrophotometer (Hitachi, U-2800) at 645, 652, 663, and 480 nm.

$$\begin{array}{c} {\mathrm{Chl}}_{a}\;(\mathrm{mg}\;g^{-}{}^{1}\;\mathrm{FW})\;=\;[12.7\;(OD663)\;-2.69\;(OD645)]V/1000\;x\;W\\ {\mathrm{Chl}}_{b}\;(\mathrm{mg}\;g^{-}{}^{1}\mathrm{FW})\;=\;[22.9\;(\mathrm{OD}\;645)\;-4.68\;(\mathrm{OD}\;663)]V/1000\;x\;W\\ {\mathrm{Chl}}_{t}\;(\mathrm{mg}\;g^{-}{}^{1}\mathrm{FW})\;=\;[20.2\;(\mathrm{OD}\;645)\;+\;8.02\;(\mathrm{OD}\;663)]V/100\;x\;W\\ \mathrm{CAR}\;(\mu g\;g^{-}{}^{1}\;\mathrm{FW})\;=\;A^{\mathrm{car}}{/E}_{\mathrm{mx}}{}^{100}. \end{array}$$

Where V is the volume of sample extract and W is the weight of the sample and A^car^ = (OD480) + 0.114 (OD663)–0.638 (OD645); E_max_^100^ cm = 2500.

### Detection of Total Free Amino Acids and Antioxidant Enzymes Activities

Fresh leaf material (1.0 g) was used to estimate total free amino acids (TFA) following the reports of Hamilton and Van Slyke (1943).

The activities of peroxidase (POX), superoxide dismutase (SOD), catalase (CAT), and ascorbate peroxidase (APX) were determined spectrophotometrically. Fresh leaf material (1 g) was homogenized in 50 mM phosphate buffer with 7.0 pH and 1 mM dithiothreitol (DTT) as described by Dixit et al. (2001). The procedure published by Zhang et al. (2012) was used to determine CAT and POX activities, whereas APX and SOD activities were measured according to the methods of Cakmak (1994) and Giannopolitis and Ries (1997), respectively.

### Determination of Shoot Se Content and Fodder Quality Attributes

The dried ground material (1 g) of above ground plant tissues (shoot and leaves) was homogenized to estimate Se accumulation in shoot using ICP-OES (Optima 2100-DV Perkin-Elmer) according to the method published in our early report (Nawaz et al., 2015b).

For fodder quality attributes, dry feeds were sampled once during each period, dried under shade for 4–5 days and milled through a 2 mm screen in a hammer mill (POLYMIX^(R)^ PX-MFC Kinematica AG Germany). Sub-samples of all feeds were analyzed for DM (Association of Official Analytical Chemists (AOAC, 1984; method 7.003), ash (525°C for 6 h; AOAC, 1984; method 923.03), CP (AOAC, 1984; method 7.015) and crude fiber (Weende method). The nitrogen free extract was calculated as %NFE = 100 – (%crude protein + %crude fat + %crude fiber + %moisture + %ash).

### Statistical Analysis

Analysis of variance (ANOVA) technique was used to statistically analyze data on STATISTIX Computer Program (Version 8.1). The significant differences among treatments’ means were compared using *post hoc* Tukey test at *P* ≤ 0.05.

## Results

### Leaf Water Status

**FIGURE 1:**
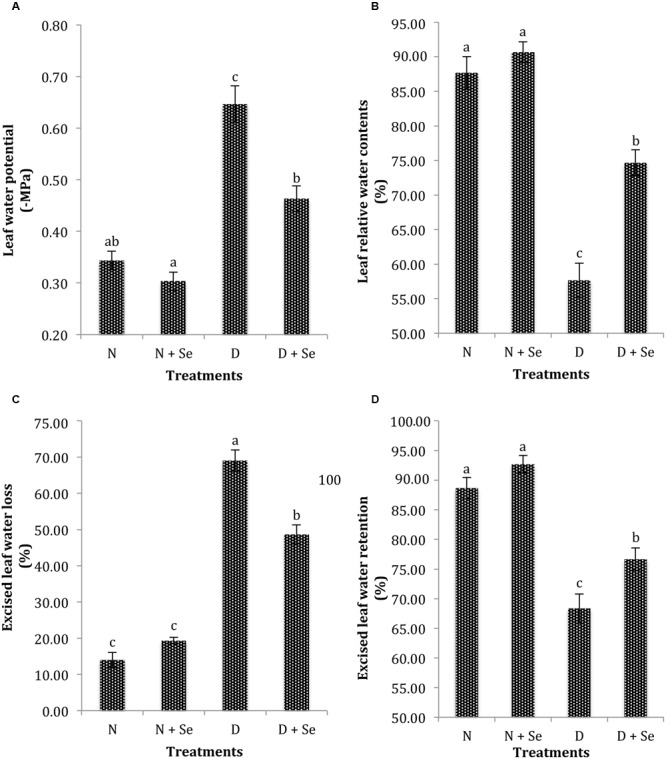
**Effect of Se foliar spray on leaf water status, **(A)** water potential (ψ_w_) **(B)** relative water contents (RWC) **(C)** excised leaf water loss (ELWL) **(D)** excised leaf water retention (ELWR) of *Zea mays* exposed to drought stress.** Values represent mean ± SE. Different letters represent significant differences at *P* ≤ 0.05, after applying *post hoc* Tukey’s test.

Drought stress (60% FC) markedly (*P* < 0.05) reduced the leaf water status of maize plants (**Table 1**). The plants supplemented with Se exhibited non-significant difference for ψ_w_ under normal conditions (100% FC), however, Se foliar spray markedly enhanced ψ_w_ by 41% with respect to no Se supply (control) under drought stress conditions (**Figure 1A**). Similar trend was noted for leaf RWC and ELWL as Se supplementation increased RWC by 30% (**Figure 1B**), whereas, it reduced ELWL by 44% (**Figure 1C**) in water stressed maize plants. Foliar Se spray also increased ELWR by 8% (**Figure 1D**) however, the interactive effects of Se × D were found to be non-significant for this variable (**Table 1**).

**Table 1:** Summary of the ANOVA for leaf water potential (WP), relative water contents (RWCs), excised leaf water loss (ELWL) and excised leaf water retention (ELWR), chlorophyll a (Chl_a_), chlorophyll b (Chl_b_), total chlorophyll (Chl_t_), and carotenoid (CAR) contents in *Zea mays*.

SOV	ψ_w_ (-MPa)	RWC (%)	ELWL (%)	ELWR (%)	Chl_a_ (mg g^-1^ FW)	Chl_b_ (mg g^-1^ FW)	Chl_t_ (mg g^-1^ FW)	CAR (μg g^-1^ FW)
Selenium (Se)	^∗∗^	^∗∗^	^∗^	^∗^	^∗∗∗^	^∗∗^	^∗∗∗^	^∗∗∗^
Drought (D)	^∗∗∗^	^∗∗∗^	^∗∗∗^	^∗∗∗^	^∗∗∗^	^∗∗∗^	^∗∗∗^	^∗∗∗^
Se × D	^∗^	^∗^	^∗∗^	NS	NS	NS	NS	^∗∗^
CV	10.03	5.04	11.44	3.59	5.27	12.34	4.21	2.65

*Significant differences are indicated by an asterisk (^∗^); ^∗^*P ≤* 0.05, ^∗∗^*P* ≤ 0.01, ^∗∗∗^*P* ≤ 0.001; NS, non-significant; SOV, source of variation; CV, coefficient of variation.*

### Pigments

**FIGURE 2:**
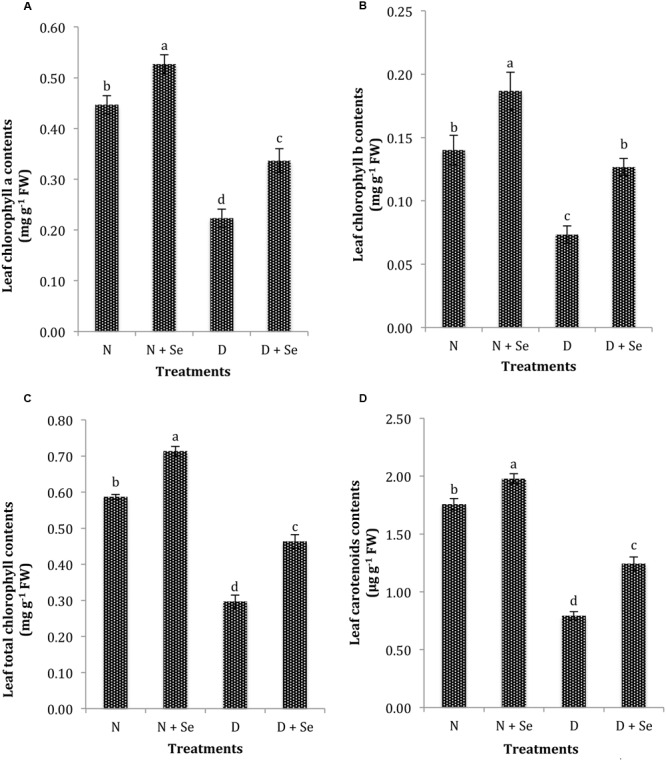
**Effect of Se foliar spray on leaf photosynthetic pigments, **(A)** chlorophyll a (Chl_a_) **(B)** chlorophyll b (Chl_b_) **(C)** total chlorophyll (Chl_t_) **(D)** carotenoid contents (CAR) of *Zea mays* exposed to drought stress.** Values represent mean ± SE. Different letters represent significant differences at *P* ≤ 0.05, after applying *post hoc* Tukey’s test.

The exposure to drought stress significantly (*P* < 0.05) reduced leaf photosynthetic pigments such as Chl_a_, Chl_b_, Chl_t_ and CAR contents by 75, 60, 71, and 84%, respectively compared to the control (100% FC). However, maize plants supplemented with Se exhibited an increase of 54, 86, 53, and 60% in leaf Chl_a_ (**Figure 2A**), Chl_b_ (**Figure 2B**), Chl_t_ (**Figure 2C**) and CAR contents (**Figure 2D**), respectively than those sprayed with water (control) under drought stress conditions.

### TFA and Antioxidant Enzymes

**FIGURE 3:**
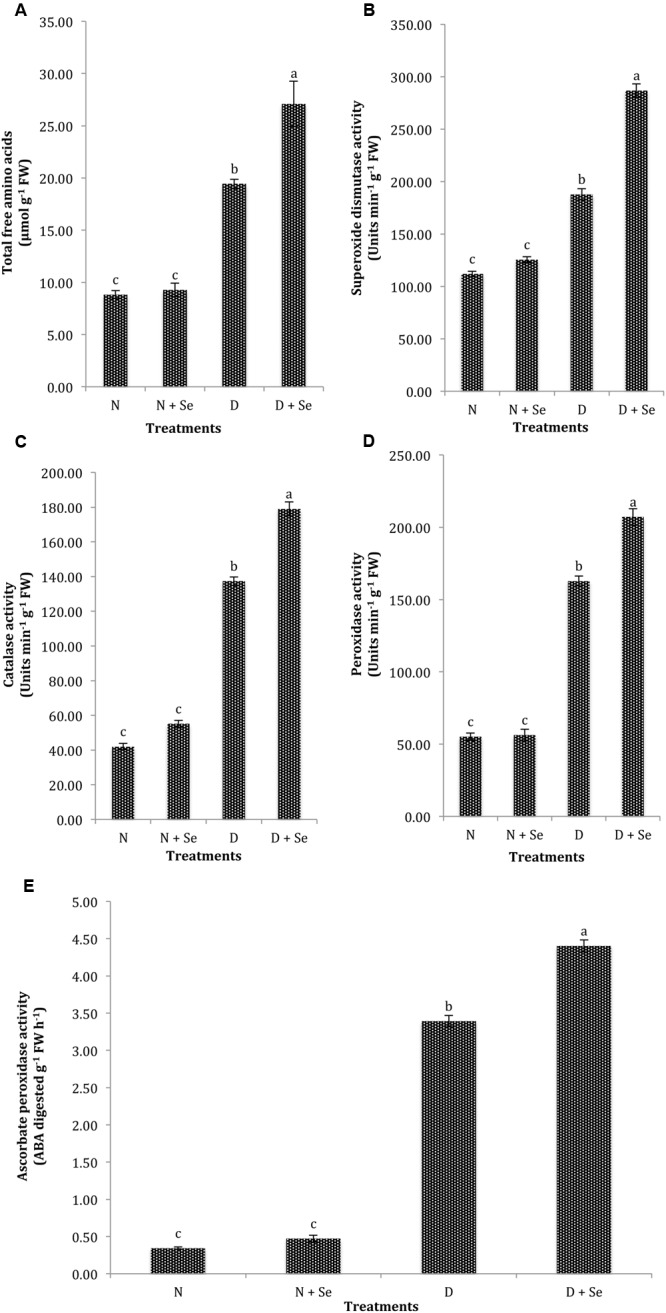
**Effect of Se foliar spray on osmoprotectants accumulation and activities of antioxidant enzymes, **(A)** total free amino acids (TFA) **(B)** superoxide dismutase (SOD) **(C)** catalase (CAT) **(D)** peroxidase (POX) **(E)** ascorbate peroxidase (APX) of *Zea mays* exposed to drought stress.** Values represent mean ± SE. Different letters represent significant differences at *P* ≤ 0.05, after applying *post hoc* Tukey’s test.

The normal plants supplemented with Se at 100% FC did not exhibit significant difference (*P* > 0.05) for the accumulation of TFA (**Table 2**), however, a marked increase (40%) in TFA content was noted by foliar Se spray in water stressed (60% FC) maize plants (**Figure 3A**). Similarly, exogenous Se supply did not significantly influence the activities of antioxidant enzymes under normal conditions. However, at 60% FC, foliar Se supplementation increased the activities of SOD, CAT, POX, and APX by 53% (**Figure 3B**), 30% (**Figure 3C**), 27% (**Figure 3D**), and 27% (**Figure 3E**), respectively with respect to control (water spray).

**Table 2:** Summary of the ANOVA for total free amino acids (TFA), superoxidase dismutase (SOD), catalase (CAT), peroxidase (POX), ascorbate peroxidase (APX), green fodder yield (GFY), and shoot Se content in *Zea mays*.

SOV	TFA (μmol g^-1^ FW)	SOD (Units min^-1^ g^-1^ FW)	CAT (Units min^-1^ g^-1^ FW)	POX (Units min^-1^ g^-1^ FW)	APX (ABA digested g^-1^ FW h^-1^)	GFY (t ha^-1^)	Se contents (μg kg^-1^ DW)
Selenium (Se)	^∗^	^∗∗∗^	^∗∗∗^	^∗∗∗^	^∗∗∗^	^∗∗^	^∗∗∗^
Drought (D)	^∗∗∗^	^∗∗∗^	^∗∗∗^	^∗∗∗^	^∗∗∗^	^∗∗∗^	NS
Se × D	^∗^	^∗∗∗^	^∗∗^	^∗∗∗^	^∗∗∗^	^∗^	NS
CV	11.91	4.08	5.01	5.23	5.15	2.60	11.70

*Significant differences are indicated by an asterisk (^∗^); ^∗^*P ≤* 0.05, ^∗∗^*P* ≤ 0.01, ^∗∗∗^*P* ≤ 0.001; NS, non-significant; SOV, source of variation; CV, coefficient of variation.*

### Fodder Quality Attributes

**FIGURE 4:**
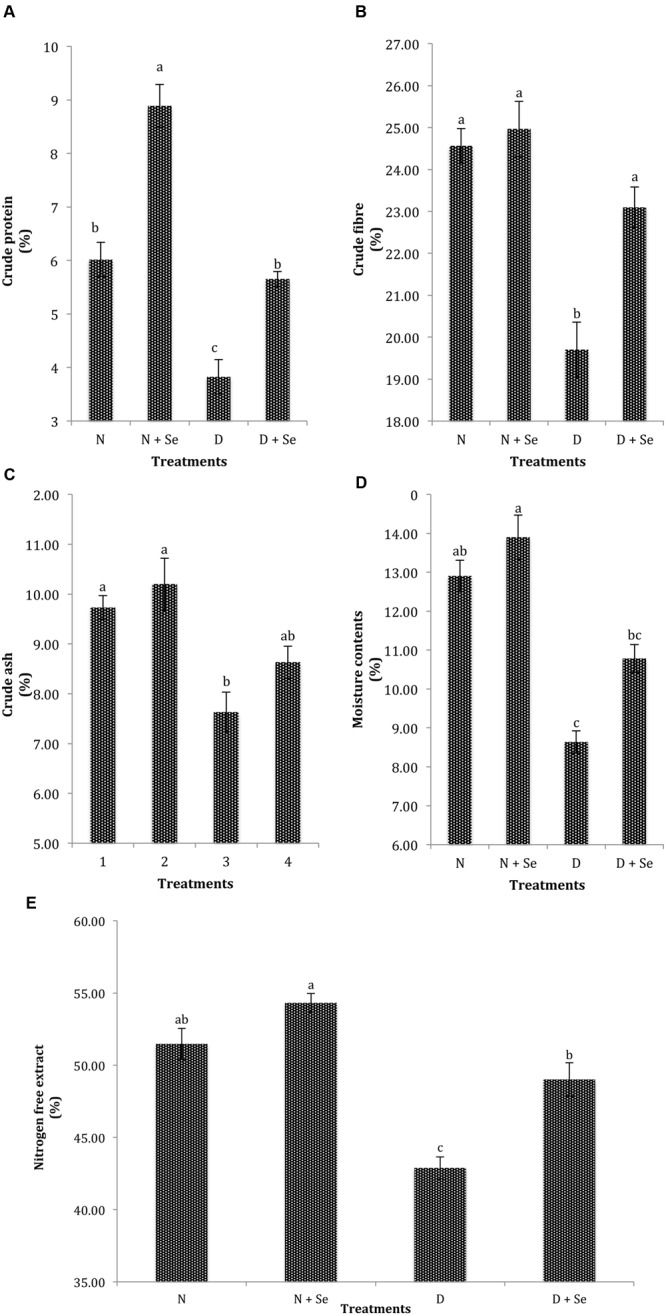
**Effect of Se foliar spray on fodder quality, **(A)** crude protein (CP) **(B)** crude fiber (CF) **(C)** crude ash (CA) **(D)** moisture contents (MC) **(E)** nitrogen free extract (NFE) of *Zea mays* exposed to drought stress.** Values represent mean ± SE. Different letters represent significant differences at *P* ≤ 0.05, after applying *post hoc* Tukey’s test.

A marked reduction of 47, 16, 23, 38, and 15% was recorded in CP, CF, CA, MC, and NFE, respectively under water deficit conditions. However, foliar Se spray significantly enhanced the quality attributes of fodder maize and increased CP, CF, MC, and NFE by 47% (**Figure 4A**), 10% (**Figure 4B**), 15% (**Figure 4D**), and 10% (**Figure 4E**), respectively but did not significantly affect CA contents (**Figure 4C**). The interactive effects (Se × D) were also found to be non-significant for fodder quality attributes (**Table 3**).

**Table 3:** Summary of the ANOVA for crude protein (CP), crude fiber (CF), crude ash (CA), moisture contents (MC), and nitrogen free extract (NFE) in *Zea mays*.

SOV	CP (%)	CF (%)	CA (%)	MC (%)	NFE (%)
Selenium (Se)	^∗^	^∗^	NS	^∗^	^∗∗^
Drought (D)	^∗∗∗^	^∗∗^	^∗∗^	^∗∗∗^	^∗∗∗^
Se × D	NS	NS	NS	NS	NS
CV	9.96	4.61	8.07	6.88	3.69

*Significant differences are indicated by an asterisk (^∗^); ^∗^*P ≤* 0.05, ^∗∗^*P* ≤ 0.01, ^∗∗∗^*P* ≤ 0.001; NS, non-significant; SOV, source of variation; CV, coefficient of variation.*

### GFY and Shoot Se Content

**FIGURE 5:**
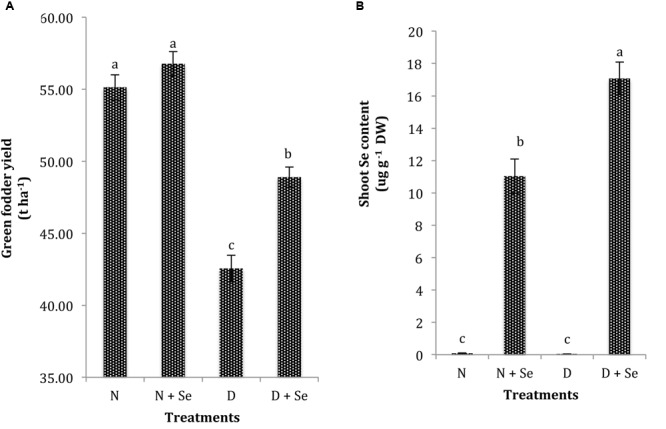
**Effect of Se foliar spray on **(A)** green fodder yield (GFY) and (B) shoot Se content of *Zea mays* exposed to drought stress.** Values represent mean ± SE. Different letters represent significant differences at *P* ≤ 0.05, after applying *post hoc* Tukey’s test.

Drought exposure (60% FC) significantly reduced the GFY of maize plants by 23% and markedly influenced the shoot Se content compared to the control (100% FC). Foliar Se supplementation was found to be effective in increasing GFY (15%) in water stressed maize plants only and did not significantly influence it under normal conditions (**Figure 5A**). Moreover, exogenous Se supply resulted in 36% higher Se contents in water stressed than normal maize plants (**Figure 5B**).

## Discussion

Maintenance of turgor through accumulation of solutes is one of the key defense mechanisms in plants to withstand the negative effects of environmental stresses particularly drought stress (Shabbir et al., 2016). The exposure to limited water supply (60% FC) markedly reduced the water status of maize plants and resulted in more negative ψ_w_ because the maintenance of favorable water relations is considered prime defense strategy of plants to tolerate drought stress (Kaldenhoff et al., 2008; Hussain et al., 2016). It was noticed that Se foliar spray resulted in less negative leaf ψ_w_ (**Table 1**), which is in agreement with the reports on potato (Germ et al., 2007), wheat (Nawaz et al., 2015a) and maize (Qiang-yun et al., 2008; Sajedi et al., 2011). The Se mediated increase in ψ_w_ might be due to its positive role in osmotic balance and ion homeostasis to increase water uptake (Kuznetsov et al., 2003) and reduce transpiration under drought stress conditions (Yao et al., 2009). The results of present study suggest that Se regulates the net accumulation of osmolytes or simple passive concentration of solutes to maintain water status in water stressed plants (Nawaz et al., 2015b). The reduction in ELWL by Se supplementation might be attributed to less negative ψ_w_ resulting in low residual transpiration (Balal et al., 2016) in under water deficit conditions. The exposure to drought stress disturbs the osmotic balance of plants that reduces turgidity and cell elongation (Shabbir et al., 2016). Previous reports have demonstrated that decrease in ψ_w_ causes a parallel reduction in RWC (Živčák et al., 2009; Raza et al., 2012; Nawaz et al., 2015a). We found that Se supplementation significantly increased the RWC of only water stressed maize plants (**Figure 1B**). The positive effects of Se supply on leaf RWC were also described by Wang (2011) and Hajiboland et al. (2014) in water stressed *Triticum aestivum* and *Trifolium repens* plants, respectively. Contrary to our results, Habibi (2013) recorded the highest RWC in Se supplemented *Hordeum vulgare* plants grown under normal conditions. The stimulating effect of exogenous Se supply may be due to increase in membrane integrity (Nawaz et al., 2015a) or decrease in photo-oxidation (Seppänen et al., 2003).

Exogenous Se supply minimizes the damage to the chloroplasts and helps to maintain photosynthetic pigments under environmental stress conditions (Zahedi et al., 2013; Khan et al., 2015; Abd-Allah et al., 2016; Balal et al., 2016). In present study, we noticed similar results in maize plants foliar applied with Se under drought stress (60% FC) conditions. Reduction in photosynthetic pigments in water stressed maize plants could have been due to chlorophyll disintegration by over production of ROS (Khayatnezhad et al., 2011). Exogenous Se supplementation influences the activities of antioxidant enzymes such as CAT, POX, APX, and SOD (also noted in present study), which help to prevent lipid peroxidation for efficient photosynthetic activity under various abiotic stresses (Habibi, 2013; Balal et al., 2016). Djanaguiraman et al. (2005) found that low Se concentrations alter chlorophyll biosynthetic pathway to increase pigments in plants. As described by Chu et al. (2010) in wheat seedlings subjected to cold stress, we found that foliar Se spray significantly enhanced the CAR contents (**Figure 2D**) in *Zea mays* exposed to water deficit conditions. Reports on *Lycium chinense* (Dong et al., 2013) and *Sorghum bicolor* (Abbas, 2012) suggest that Se supplementation enhances photosynthetic pigments at low doses but causes a marked reduction at high doses due to negative effect on the production of porphobilinogen synthetase (Padmaja et al., 1995) or replacement of sulfur (S) atoms by Se in S-containing amino acids, cysteine and methionine (Terry et al., 2000). However, Hawrylak-Nowak (2009) observed non-significant effect of Se supplementation on CAR contents of cucumber plants. Se mediated increase in CAR pigments further supports the theory that CAR pigments are involved in scavenging of free radicals and maintenance of membrane integrity (Peng and Zhou, 2009).

Foliar Se spray significantly increased the accumulation of osmotically active molecules like TFA (**Figure 3A**), which helped drought stressed maize plants to maintain water status. It can be inferred from the results that Se stimulates amylase activity to increase starch decomposition under water deficit conditions. Early reports on *Glycine max, Solanum tuberosum, Brassica napus, and Zizyphus jujube* (Djanaguiraman et al., 2004; Turakainen et al., 2004; Hajiboland and Keivanfar, 2012; Zhao et al., 2013) are concordant with our results suggesting that exposure to environmental stresses like drought results in the breakdown of structural proteins to promote biosynthesis and accumulation of amino acids (Hsu et al., 2003), which actively take part in osmotic adjustment under drought stress conditions (Good and Zaplachinski, 1994). Djanaguiraman et al. (2004) were of the view that Se supplementation disturbs the amino acid metabolism, which increases soluble protein content and nitrate reductase activity in water stressed plants. The increased activities of antioxidant enzymes indicate excessive ROS production under drought stress conditions (Cartes et al., 2010; Hasanuzzaman and Fujita, 2011). These enzymes serve as highly efficient machinery for detoxification of O_2_^-^ and H_2_O_2_ and help to prevent the formation of highly toxic HO^-^ (Mittler et al., 2004). A marked increase in SOD, CAT, POX, and APX production by Se supplementation provides further evidence that Se regulates the spontaneous dismutation of O_2_^-^ into H_2_O_2_ (Hartikainen et al., 2000; Cartes et al., 2010) or may be directly involved in quenching of O_2_^-^ and OH^-^ in cells (Xu et al., 2007). Previous studies in wheat (Yao et al., 2009; Nawaz et al., 2015a), barley (Habibi, 2013), tomato (Balal et al., 2016), and rice (Xu and Hu, 2004) also reported an increase in the activity of antioxidant machinery in Se supplemented plants exposed to a wide range of abiotic stresses. It is crucial to maintain balance between SOD and other ROS scavenging enzymes to determine the steady-state level of O_2_^-^ and H_2_O_2_ in cells (Mittler et al., 2004) hence the exogenous application of appropriate doses of Se is involved in the reactivation of ROS quenchers like SOD, POX, and GSH-Px to reduce H_2_O_2_ levels in plants exposed to stressful environments (Filek et al., 2009; Kumar et al., 2012). Earlier, Sajedi et al. (2011) reported increased antioxidant activity in Se supplemented maize plants and suggested that single but not the combined use of Se or micronutrients mitigates drought stress in plants. However, the excessive Se doses not only reduce the POX activities (Nowak et al., 2004) but also cause damage to plant tissues (Marschner, 1995; Nawaz et al., 2013).

Drought induced reduction in maize fodder yield and quality corresponds to the reports in sorghum-sudan grass (Bibi et al., 2012) and ryegrass (Abraha et al., 2015). Positive effects of Se on yield of food crops are well-documented (Ducsay et al., 2009; Chilimba et al., 2012; Wang et al., 2013; Nawaz et al., 2015b), however, reports regarding role of Se in improving forages yield are scanty. Availability, of high soil moisture content is one of most critical factors influencing the yield of forages in water-limited environment (Staniak and Kocoń, 2015). Presumably, Se mediated increase in maize fodder yield is related to the maintenance of turgor and enhancement in photosynthetic pigments that helped plants to produce more biomass under water deficit conditions. Foliar Se application significantly increased the fodder quality attributes such as CP, CF, and NFE which might be attributed to improved water status and increased activity of antioxidant machinery that would have stimulated uptake of minerals and translocation of assimilates to shoot which enhanced fodder quality. Our results are concordant with the reports of Pii et al. (2015) who were of the view that physiological processes and biochemical activities including enzyme activation control the elemental uptake that defines the nutritional status of plants. However, interactions between Se and other elements are also well-reported in literature for example; Yao et al. (2013) documented that exogenous Se supply significantly enhanced the uptake of Fe, K and Zn in wheat, whereas, Pazurkiewicz-Kocot et al. (2008) noticed that Se supplementation increased the K contents in maize grains but inhibited the accumulation of Ca and Mg in roots. Recently, Drahoňovský et al. (2016) observed marked increase in Ca contents of Se supplemented *Chaerophyllum temulum* and *Veronica chamaedrys*, hence, further targeted research is necessary to investigate the relation between Se supply and nutrients uptake and its association with fodder quality attributes. Increased Se content in maize plants supports our hypothesis that foliar Se supply can be utilized as an effective strategy for the biofortification of fodder crops (Drahoňovský et al., 2016) to prepare balanced mineral ration for livestock in areas with low Se levels in pastures (Žáková, 2014). Higher Se contents in water stressed plants might be due to enhanced activities of antioxidant enzymes with respect to control (Hartikainen et al., 2000). Cartes et al. (2005) documented that a positive correlation exists between shoot Se concentration and GSH-Px activity, which might be responsible for increased shoot Se content under water deficit conditions. Our results further support the notion that soil moisture influences Se availability and accumulation, as it is more available under limited water environment (Zhao et al., 2007).

## Conclusion

To the best of our knowledge, the present study is one of the few reports on the effects of exogenous Se supply on maize fodder yield and quality under water deficit conditions. Positive effects of Se supplementation on fodder yield and quality were found to be associated with Se-mediated regulation of physiological and biochemical processes such as maintenance of turgor due to accumulation of osmolytes like TFA, increased chlorophyll and carotenoid contents and activation of antioxidant machinery in water stressed maize plants. Moreover, foliar Se supply also increased Se content in shoot, which may be exploited as a viable and effective approach to increase Se concentration in fodders for the development of balanced livestock ration particularly in areas with low Se levels in soils.

## Author Contributions

FN, MN, and MT conceived and designed the study, FN wrote the manuscript, MA and RS provided reagents and analyzed results, BZ and MS performed the analytical works, MA managed the treatments application.

## Conflict of Interest Statement

The authors declare that the research was conducted in the absence of any commercial or financial relationships that could be construed as a potential conflict of interest.
